# 고강도 집속 초음파 치료를 받는 자궁양성종양 환자의 동영상 교육프로그램 효과

**DOI:** 10.4069/kjwhn.2020.03.31

**Published:** 2020-06-24

**Authors:** Mi Suk Hong, Hyoung Sook Park, Young Suk Cho

**Affiliations:** 1 Changwon Jeil General Hospital, Changwon, Korea; 1 창원제일종합병원 간호부; 2 College of Nursing, Pusan National University, Yangsan, Korea; 2 부산대학교 간호대학; 3 Korean Nursing Educational Credentialing Center, Korean Nurses Association, Seoul, Korea; 3 한국간호평가원, 한국간호평가원, 대한간호협회

**Keywords:** Emotions, Self efficacy, Uncertainty, Uterine neoplasms, 기분, 자기효능감, 불확실성, 자궁양성종양

## Abstract

**Purpose:**

The purpose of this study was to examine the effects of a video education program in women receiving high-intensity focused ultrasound (HIFU) treatment.

**Methods:**

This was a quasi-experimental study with a nonequivalent control group non-synchronized design. The participants were 54 patients who had benign uterine tumors and adenomyosis. The data were collected from June to August 2018. A 10-minute video education program on HIFU and post-procedural care was developed based on the literature. The experimental group was provided the video education program with a question-and-answer session for 10 minutes after viewing the video. The control group received usual care (i.e., verbal instructions on post-procedural self-care). The questionnaire survey was conducted twice: before the educational program and before being discharged from the hospital. Differences in uncertainty, emotions, and self-efficacy among patients were analyzed. Data were analyzed using the chi-square test, Shapiro-Wilk test, paired t-test, and t-test with SPSS version 23.0.

**Results:**

The participants in the experimental group showed a decrease in uncertainty(t=4.33, *p*<.001), improvements in anxiety(t=–4.07, *p*<.001) and depression (t=–3.55,*p*<.001), and an enhancement ofself-efficacy (t=–4.39,*p*<.001) compared to the control group.

**Conclusion:**

This nursing intervention was effective at reducing uncertainty, improving emotions, and enhancing self-efficacy. This intervention is feasible for use in nursing practice as an aid for patients when considering treatment methods.

## Introduction

### 연구 필요성

한국 여성의 자궁절제술은 연간 총 4만 5천건으로 여성 생식기 질환 수술 중 1위를 차지하고 있으며[1], 세계경제협력개발기구(Organization for Economic Cooperation and Development, OECD)의 건강 통계자료에 의하면 OECD 평균 건수보다 3배나 많아 아시아 국가 중 1위를 기록하고 있다[2]. 자궁절제술을 받은 여성 환자들은 수술 후 월경이 없어지고 임신이 불가능해지는 것을 여성성의 상실로 받아들여 수술 후 불안과 우울, 성 역할 정체성 및 삶의 질 변화에 따른 정서적인 반응장애 등의 다양한 어려움을 경험한다[3].

이러한 문제로 인하여 자궁절제술의 대안으로 비수술적 치료법인 고강도 집속 초음파(high intensity focused ultrasound, HIFU) 치료가 도입되었다. 인체 외부에서 고강도 초음파로 병변을 태워 괴사시키는 HIFU 치료의 효과는 2004년 미국 식약청[4]으로부터 승인을 받았으며, 한국의 경우 보건복지부가 2008년 신의료기술로 인정한[5] 이후 현재까지 자궁양성종양의 치료방법으로 적용하고 있다. HIFU 치료의 성공률은 약물 요법인 미페프리스톤(mifepristone)보다 상당히 높고, 전통적으로 실시되어 오고 있는 자궁근종 절제술 또는 자궁절제술에 필적한다[6]. 치료의 안정성을 비교하면 미페프리스톤, 자궁근종 절제술 및 자궁절제술보다 치료 후 통증, 불쾌감, 발열, 감염, 생식기계, 위장관계 및 마취와 관련된 합병증이 거의 없고 치료과정에서 출혈이 없어 수혈의 위험이 없음이 보고됨에 따라[6], 가임기 여성이 아니더라도 자궁 적출을 원하지 않는 여성들이 HIFU 치료를 선택함으로써 자궁 적출에 따른 신체적 ·정신적 부담감으로부터 자유로워지고 있다.

HIFU 치료를 받는 환자들은 전신마취와 의식 하 진정 등이 없는 상황에서 치료를 받으므로, 치료 전 의료진으로부터 정보를 제공받았음에도 불구하고 커다란 장비 위에 엎드린 자세를 취하고 차가운 물에 복부를 담근 채 1시간 이상의 긴 시간을 꼼짝도 못하고 누워 있어야 하는 낯선 환경에 직면한다. 또한 HIFU 치료 중 복부 장기의 움직임을 방지하기 위해 복부를 압박하는 물주머니와 유치도뇨관을 삽입해 방광을 인위적으로 팽만시켜 복부를 압박한 상태에서 자궁양성종양인 자궁근종과 자궁선근증의 병변에 고강도 초음파를 가하면서 통증이 발생하는 등 익숙하지 않은 치료과정으로 인해 불안을 경험한다[7]

그러나 Kang과 Park [8]은 HIFU 치료에 대한 정확한 지침이 없다는 연구의 제한점을 제시하였다. Park과Kim [7] 또한 HIFU 치료를 받는 환자들이 치료 전 의료진으로부터 설명을 들었음에도 불구하고 정보의 누락이나 교육 내용이 실제 환경과 맞지 않음 등의 지식 부족으로 인해 치료과정에서 불안, 불확실성, 통증 및 공포 등을 경험하므로 HIFU 치료를 받는 환자의 불안과 불확실성을 완화시켜 줄 수 있는 간호 중재 프로그램의 개발이 필요하다고 제안하였다.

수술을 앞둔 환자는 막연한 두려움과 걱정으로 불안하며, 치료과정에 대한 이해가 부족한 경우 치료상황을 위협으로 받아들여 부적응을 초래한다. 이로 인해 불확실성, 불안, 우울이 가중되면서 자기 효능감이 떨어져 치료과정에 적극 참여하지 못하게 된다[9-11]. 선행연구를 보면 간호사로부터 간호 정보를 제공받은 환자들은 자신들의 질병 상태에 관한 정보를 습득하면서 불확실성[12], 불안 및 우울이 감소되어 기분이 안정되고[13,14] 자신의 치료과정에 적극 참여하게 되어 자기 효능감도 강화되었다[15]고 한다. 그러므로 의식이 명료한 상태로 낯선 기계와 병원 환경에 둘러싸여 1시간 반에서 2시간 가량의 장시간 치료를 받으며 불안, 불확실성, 통증 및 공포를 경험하는[7] HIFU 치료 환자들에게, 치료 공간에 함께 있는 간호사의 정보 제공, 간호 중재 및 정신적인 지지는 매우 중요한 역할을 할 것이다.

국내외에서는 HIFU 치료 전 교육, 증상 관리 및 퇴원 교육 등을 모두 간호사가 주도적으로 수행해 왔다. 그러나 전립선 암 환자 대상 HIFU 치료에 대한 간호사의 역할에 대한 연구[16]와 자궁양성종양 환자 대상 HIFU 치료 후의 운동 중재 효과를 검정한 연구[17]를 제외하면, 한국은 HIFU 치료가 실시된 이후 현재까지 HIFU 치료 환자의 경험을 연구한 질적 연구[7]가 있을 뿐, 간호사가 개발한 간호 중재 또는 간호 교육을 제공한 후 그 효과를 검정한 선행연구는 거의 찾아볼 수 없다.

최근 의료 환경은 마취와 절개를 행해야 하는 전통적인 수술법보다 전신마취와 절개를 하지 않는 최소 침습 또는 비침습적인 방법으로 환자의 안위를 증진시키고 치료로 인한 사회생활의 지장을 최소화하는 방향으로 점점 발전하고 있다. 자궁양성종양 치료 분야 내에서도 HIFU 치료는 자궁을 그대로 보존하며 사회생활에 지장이 없는 비수술적 치료를 원하는 자궁양성종양 환자들이 선호하는 치료법으로, 더욱 발전하고 많은 환자에게 적용될 수 밖에 없는 치료이다.

이에 본 연구자들은 자궁양성종양 환자를 대상으로 HIFU 치료의 방법, 치료 전 환자의 준비과정으로 금식, 제모, 관장, 유치도뇨관 삽입의 필요성과 중요성을 살펴보고, 치료과정 및 퇴원 후 전반적인 추후 관리 중재를 위해 동영상 교육프로그램을 개발·적용하여 HIFU 치료를 받는 환자의 불확실성, 기분 및 자기 효능감에 미치는 효과를 검정하고 이러한 환자들의 적응을 향상시키고자 본 연구를 수행하였다. 이는 궁극적으로 HIFU 치료를 받는 환자들을 위한 간호사의 적극적인 활동으로 간호 중재를 질적으로 향상시키는 동시에 간호행위별 수가 개발을 위한 기초자료를 제공하는데 기여할 것이다.

### 연구 목적

본 연구의 목적은 HIFU 치료를 받는 대상자에게 동영상 교육프로그램을 제공하고 그 효과를 검정하는 것으로, 연구가설은 다음과 같다.

• 가설 1. 동영상 교육프로그램을 제공받은 실험군은 제공받지 않은 대조군보다 불확실성이 감소할 것이다.

• 가설 2. 동영상 교육프로그램을 제공받은 실험군은 제공받지 않은 대조군보다 기분이 개선될 것이다.

• 가설 3. 동영상 교육프로그램을 제공받은 실험군은 제공받지 않은 대조군보다 자기 효능감이 향상될 것이다.

## Methods

Ethics statement: This study was approved by the Institutional Review Board of Changshin University (CSIRB-R20180006). Informed consent was obtained from the participants.

### 연구 설계

**Figure 1.**
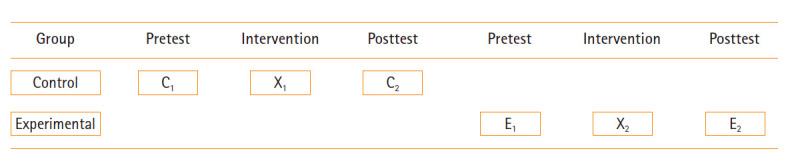
The study design. C1, E1: General characteristics, disease-related characteristics, uncertainty, emotions, self-efficacy; C2, E2: uncertainty, emotions, self-efficacy; X1: usual care (hospitalization manual, safety accident prevention guide, oral description of preparation and treatment process); X2: video-based education program.

본 연구는 HIFU 치료 전 연구 대상자에게 동영상 교육프로그램을 제공한 후 그 효과를 분석하기 위한 비동등성 대조군 전후 시차설계의 유사 실험연구이다(Figure 1).

대조군은 2018년 6월 23일부터 7월 22일까지 1개월간 조사하였고, 2018년 7월 23일부터 8월 22일까지 1개월간의 치료 환자를 실험군으로 선정하였다. 시차설계를 적용함으로써 실험의 확산을 방지하고 연구 대상자가 자신이 실험군인지 대조군인지 알 수 없도록 이중차단장치 방법을 적용하였다. 연구 대상자들에게 사전에 연구의 목적을 설명하고 연구 참여의 이익과 위험을 충분히 설명한 후, 본 연구에 서면 동의한 환자를 대상으로 실시하였다.

### 연구 대상

본 연구의 대상자는 창원시 소재 종합병원 산부인과 병동에 자궁양성종양으로 HIFU 치료를 받기 위해 입원한 환자로, 대상자의 선정기준은 첫째, 본 연구의 목적과 방법을 이해하고 연구 참여에 동의한 환자, 둘째, 양성종양의 크기와 관계없이 진단명이 자궁근종 또는 자궁선근증인 환자, 셋째, 언어적∙비언어적인 의사소통이 가능한 환자이다. 제외기준은 첫째, 한국어를 읽고 이해하는데 어려움이 있는 외국인 환자, 둘째, 한국어로 된 동영상을 시청하고 이해하는 데 어려움이 있는 외국인 환자는 연구대상에서 제외하였다.

본 연구 대상자들이 치료를 받은 HIFU 기종은 엎드린 자세(prone position)로 시술받는 초음파 유도하(ultrasound guided) HIFU인 JC-2000 (Haifu Technology, Chongqing, China)과 자기공명 유도하(magnetic resonance guied) HIFU인 Philips Sonalleve (Philips Healthcare, Vantaa, Finland)이다.

**Figure 2.**
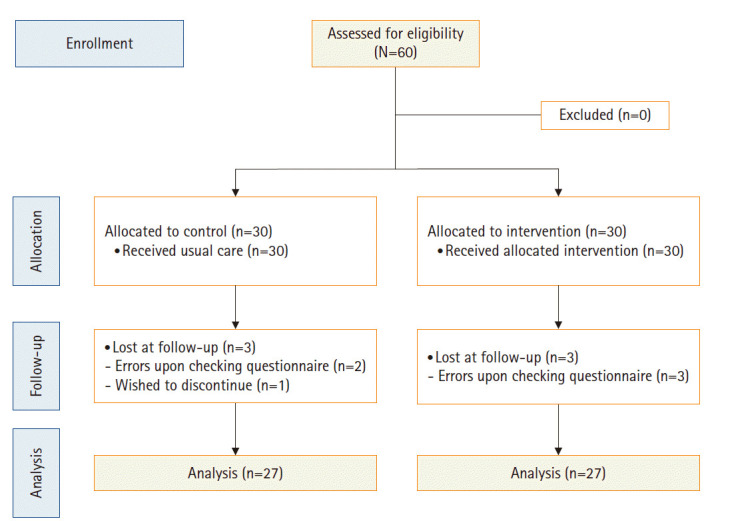
Flow chart of this study.

표본의 크기는 HIFU 치료 전 동영상 교육프로그램의 적용 효과에 대한 선행연구를 찾기 어려워, 자궁근종 환자를 대상으로 동영상 교육프로그램을 실시한 Eo 등[14]과 Jeon과 Park [18]의 공식에 근거한 효과 크기(d) 0.5, 검정력(1–β) .80, 단측검정 유의수준(α) .05를 기준으로 실험군과 대조군 각각 27명으로 총 54명을 산출하였다. 자궁근종 환자를 대상으로 동영상 교육프로그램을 실시한 선행연구에서 중도 탈락률이 10.0% 미만이었던 결과를 감안하여[13,19] 초기 연구 대상자의 중도 탈락률을 10.0%로 고려하고 총 60명의 자료를 수집하였다. 연구가 진행되는 동안 실험군의 설문지 체크 오류가 발생한 3명, 대조군 중 피곤하여 연구 참여 철회를 희망한 1명과 설문지 체크 오류가 발생한 2명이 탈락하여 최종 분석 대상은 실험군 27명, 대조군 27명으로 전체 54명이었다(Figure 2).

### 연구 도구

#### 불확실성

연구 대상자의 불확실성을 측정하기 위하여 Mishel [10]이 개발하고 Chung 등[20]이 한국어로 번역한 불확실성 척도(Mishel Uncertainty in Illness, MUIS)를 사용하였다. 연구에 사용된 도구는 원저자와 한국판 저자로부터 이메일을 통해 사용 허락을 받았다. 33문항 중 15번 ‘의료진이 너무 많아 누가 무엇을 책임지는지 알 수 없다’ 문항은 HIFU 치료 의료진이 명확하여 본 연구의 질문으로 부적절하므로 제외하고 32문항으로 수정하여, 간호학 교수 1인, HIFU 센터 수간호사 1인의 타당도 검정을 받은 후 사용하였다.

각 문항은 1점에서 5점의 평정척도로 ‘매우 그렇지 않다’를 1점, ‘매우 그렇다’를 5점으로 평정하였고, 총점은 모든 문항의 점수를 합하여 최저 32점, 최고 160점의 범위를 가지며 점수가 높을수록 불확실성이 큰 것을 의미한다. Mishel [10] 연구의 신뢰도(Cronbach’s α)는 .85, Chung 등[18]의 Cronbach’s α는 .87이었고, 본 연구의 Cronbach’s α는 .86이었다.

#### 기분

연구 대상자의 기분 정도를 측정하기 위하여 병원을 방문한 환자가 의사의 진료를 받기 위해 외래와 병실에서 기다리는 동안 가장 흔히 느끼는 환자 불안과 우울을 측정한 Zigmond와 Snaith [21]의 척도를 Oh 등[11]이 한국어로 번역한 도구를 사용하였다. 연구에 사용된 도구는 도구 사용료를 지불(www.gl-assessment.co.uk)했으며, 한국판 저자로부터 이메일을 통해 사용 허락을 받았다. 이 도구는 환자 불안과 우울 각 7문항으로 구성되어 총 14문항이다. 각 문항은 1점에서 4점의 평정척도로 총점은 모든 문항의 점수를 합하여 최저 14점, 최고 56점의 범위를 가지며 점수가 높을수록 불안과 우울 정도가 높아 기분이 나쁜 것을 의미한다. Zigmond와 Snaith [21] 연구의 Cronbach’s α는 .85, Oh 등[11]의 Cronbach’s α는 .89이었고, 본 연구의 Cronbach’s α는 .93이었다.

#### 자기 효능감

연구 대상자의 HIFU 치료 후 자가간호에 관한 자기 효능감을 측정하기 위하여 Hong [22]이 개발한 자기 효능감 측정도구를 Park [23]이 수정·보완한 도구를 사용하였다. 이 도구는 총 12문항으로 구성되어 있으며 각 문항은 1점에서 5점의 평정척도로 ‘매우 자신이 없다’를 1점, ‘매우 자신이 있다’를 5점으로 평정하고, 총점은 각 문항의 점수를 합하여 최저 12점, 최고 60점의 범위를 가지며 점수가 높을수록 자기 효능감이 높음을 의미한다. Park [23] 연구의 Cronbach’s α는 .83이었고, 본 연구의 Cronbach’s α 역시 .83이었다.

#### 일반적 특성과 질병 관련 특성

연구 대상자의 일반적 특성은 연령, 결혼 상태, 교육 수준, 직업 및 경제 상태 등의 총 5개 문항이었고, 질병 관련 특성은 진단명과 HIFU 치료 정보를 얻은 경로 등의 총 2개 문항이었다.

### HIFU 치료 동영상 교육프로그램 개발과정

원내 HIFU 시술을 하는 센터 실무자가 참여하여 교육 내용을 구성하였다. 교육 내용의 구성은 HIFU 센터와 산부인과 병동에서 사용하고 있는 HIFU 치료 관련 교육 자료, HIFU 기기별 치료 프로토콜과 권고안[25], 환자들의 치료 후기, 여성건강 간호학 및 의학 교재인 부인과학[26], HIFU 전문의의 의견 등등을 참고로 하였으며, HIFU 치료를 받은 환자들에게 HIFU 치료와 관련하여 교육받기를 원하는 내용을 개방형으로 진술하게 하고 이 진술 내용을 포함시켜 교육 내용을 더욱 풍부하게 하였다.

동영상의 주요 교육 내용은 자궁양성종양의 치료적인 특성, 자궁적출술이 여성 건강에 미치는 영향, 자궁과 여성 생식기의 해부학적 위치와 생리, 자궁의 인체 내 기능, HIFU 치료의 원리, HIFU 치료의 적응 대상, HIFU 치료법의 안전성, 안전한 HIFU 치료를 위한 사전 검사 항목과 복부 자기공명영상 촬영법에 관한 설명, 제모, 관장 및 유치도뇨관의 필요성, 치료과정의 자세, 의사소통, 통증 발생 시 처치에 관련된 설명, HIFU 치료 후 나타날 수 있는 증상, 샤워 시기, 퇴원 후 식이조절, 운동, 성생활 및 임신 가능한 시기에 관한 주의사항 등등에 관한 것이었다(Table 1).

교육 내용을 구성한 후 본 연구의 대상자는 아니지만 자궁양성종양을 진단받은 환자 2명에게 교육 자료를 시청하게 한 후 어려운 의학용어나 이해되지 않는 부분을 검토 받아 그 내용을 수정하였다. 수정된 내용을 바탕으로 사진, 동영상 및 파워포인트 자료 등을 이용하여 교육 자료를 구성한 후, 애니메이션을 활용한 교육 자료에 대한 환자들의 수용도가 높은 점을 감안하여 애니메이션을 이용하여 나레이션과 자막 처리하였으며, 제작기간은 총 2개월이 소요되었다.

제작된 동영상의 구성 및 내용의 타당도를 높이기 위하여 간호학 교수, 산부인과 의사 1인, HIFU 센터 수간호사 1인에게 타당도 검정을 받은 후 이용하였으며 교육용 동영상은 선행 자궁적출술 환자들에게 적용되었던 교육프로그램을 분석하여 “현명한 나의 선택, HIFU”라는 제목으로 총 10분 분량으로 제작되었다.

### 연구진행절차

#### 사전 조사

사전 조사는 실험군과 대조군을 대상으로 이루어졌다. 부인과 수술 환자를 대상으로 불안 정도를 측정한 결과 수술 전일 18:00시부터 점차 상승하여 마취 전 불안이 가장 높았고 수술 후 1일째부터는 불안이 감소하였다고 보고한 Carr 등[24]의 연구 결과를 근거로 사전·사후 설문조사 시점을 정하였다. 예약 당일 입원하며 입원 당일 치료에 필요한 검사와 처치 후 치료를 진행하는 HIFU 치료의 특성상, HIFU 치료를 받기 위해 병원을 방문한 당일 입원 수속 후 불확실성, 기분 및 자기 효능감 설문지를 이용하여 본 연구자가 조사하였다. 설문지 작성은 연구 대상자가 스스로 읽고 직접 기입하였다.

#### 실험 처치

실험군에게는 태블릿 PC를 이용하여 HIFU 치료 전 동영상 교육프로그램을 제공하였다. 처치의 효과를 극대화하기 위해 동영상 교육프로그램은 연구자가 직접 제작하여 교육을 실시하였다. 실험 처치에 소요한 시간은 동영상 시청 10분과 이해되지 않는 부분에 관한 질의응답 및 부연 설명까지 총 20분간이며, HIFU 치료 전 1회 제공되었다.

#### 사후 조사

사후 조사는 실험군에게 HIFU 치료 전 동영상 교육프로그램을 제공한 후 HIFU 치료를 받은 다음 날 아침 사전 조사에 사용한 동일한 설문지를 통해 불확실성, 기분 및 자기 효능감의 자료를 수집하였다. 대조군은 HIFU 치료 전 동영상 교육프로그램을 제공하지 않고 구두로 기존의 HIFU 치료 전 준비과정과 치료과정 등의 정보를 제공한 후 HIFU 치료를 받은 다음 날 아침 불확실성, 기분 및 자기 효능감에 관한 설문지를 작성하게 하였다.

대조군의 경우 사후 설문조사가 끝난 시점에 동영상 교육프로그램을 제공하고 동영상 시청이 끝난 후 10분 동안 상담을 제공하여 대조군의 윤리적 불이익을 최소화하도록 하였다. 모든 연구 대상자에게는 소정의 답례품으로 핸드크림 세트를 제공하였다.

### 자료 분석

수집된 자료는 IBM SPSS Statistics for Windows, ver. 23.0 (IBM Corp., Armonk, NY, USA)을 이용하여 다음과 같이 분석하였다.

1) 연구 대상자의 일반적 특성과 질병 관련 특성은 실수와 백분율, 평균과 표준편차를 구하였다.

2) 연구 대상자의 일반적 특성과 질병 관련 특성에 따른 동질성 검정은 chi-square test, t-test를 이용하였고, 종속변수의 동질성 검정은 t-test로 분석하였다.

3) 실험군과 대조군의 각 변수에 대한 정규성 검정은 Shapiro-Wilk test를 통해 유의수준(α) .05를 기준으로 검정하였다.

4) 실험군의 동영상 기반 교육프로그램의 HIFU 치료 전 적용 전 · 후 차이는 paired t-test, 실험군과 대조군의 실험 처치 후 가설검정은 t-test로 분석하였다. 유의수준(α) =.05, 단측검정으로 분석하고 *p*<.025이면 유의성이 있다고 해석한다.

## Results

### 연구 대상자의 동질성 검정

연구 대상자의 일반적 특성과 질병 관련 특성에 대한 동질성 차이 검정의 결과는 Table 1과 같다.

전체 연구 대상자의 평균 연령은 44.16±6.58세였고, 79.6%가 기혼으로 나타났다. 68.5%가 대졸 이상이었으며, 72.2%는 직장을 다니지 않았고, 경제 상태는 89.0%가 중, 5.5%는 상으로 나타나 중상층의 비율이 높았다. 실험군과 대조군의 연령, 결혼 상태, 교육 수준, 직업 및 경제 상태 등에 대해 통계적으로 유의한 차이가 없는 것으로 나타나 두 집단은 유사한 집단임을 확인하였다(Table 2).

연구 대상자의 진단명은 자궁근종이 70.4%를 차지하였다. HIFU 치료의 정보를 획득한 경로는 인터넷이 70.4%로 가장 많았으며, 산부인과 전문의로부터 설명을 들은 환자는 5.5%였다. 실험군과 대조군의 질병 관련 특성인 진단명 및 HIFU 치료 정보를 얻은 경로 등에 대해 통계적으로 유의한 차이가 없는 것으로 나타나 두 집단은 유사한 집단임을 확인하였다(Table 2).

실험군과 대조군의 연구 대상자 표본은 각각 27명으로 연구변수에 대한 정규성 검정을 위해 Shapiro-Wilk test를 실시한 결과 불확실성, 기분 및 자기 효능감은 모두 정규성을 따르는 것으로 나타났다(Table 3).

### 가설 검정

동영상 교육프로그램을 제공받은 실험군과 대조군의 불확실성 변화를 분석한 결과, 실험군의 불확실성 점수가 14.85±17.47점 감소하였고 대조군은 3.19±9.66점 감소하여 통계적으로 유의하였다(t=4.33, *p*<.001). 따라서 ‘동영상 교육프로그램을 제공받은 실험군은 제공받지 않은 대조군보다 불확실성이 감소할 것이다’라는 제1가설은 지지되었다(Table 3 ).

동영상 교육프로그램을 제공받은 실험군과 대조군의 기분 변화는 실험군의 불안 점수가 4.44±4.33점, 대조군은 0.11±1.21점 감소하였고(t=–4.07, *p*<.001), 우울 점수는 실험군이 2.67±3.55점, 대조군은 0.33±2.05점 감소를 보여(t=–3.55, *p*<.001), 통계적으로 유의하였다. 따라서 ‘동영상 교육프로그램을 제공받은 실험군은 제공받지 않은 대조군보다 기분이 개선될 것이다’라는 제2가설은 지지되었다(Table 3).

동영상 교육프로그램을 제공받은 실험군과 대조군의 자기 효능감 변화를 분석한 결과, 실험군의 자기 효능감 점수는 4.18±4.72점, 대조군은 0.51±2.95점 증가하여 통계적으로 유의하였다(t=–4.39, *p*<.001). 따라서 제3가설 ‘동영상 교육프로그램을 제공받은 실험군은 제공받지 않은 대조군보다 자기 효능감이 향상될 것이다’ 역시 지지되었다(Table 3).

## Discussion

본 연구는 HIFU 치료를 받는 자궁양성종양 환자에게 동영상 교육프로그램을 제공한 후 동영상 교육프로그램의 효과를 검정하고자 수행된 연구로써 그 결과를 중심으로 논의하고자 한다.

본 연구의 동영상 교육프로그램은 HIFU 치료를 받는 자궁양성종양 환자의 불확실성을 감소시키는 데 효과적이었다. HIFU 치료를 받는 환자들은 수술을 제외한 약물과 다양한 대증요법 등의 치료를 받아 보았지만 경과가 불량했던 경험으로 인해, HIFU 치료 역시 수술하지 않는 새로운 치료법이라 여기는 불확실성을 유발할 수 있다. 또한 HIFU 치료는 새로운 의료기술이므로 환자의 주변에 HIFU 치료를 받은 사람이 없는 경우가 많다. 그러므로 치료과정에 관한 경험을 듣지 못하여, 마취 없이 치료가 이루어지는 HIFU 치료 중 초음파 열이 가해질 때의 통증에 대한 불안, 통증으로 인해 치료를 끝까지 받지 못할 우려 등 불확실성이 가중되기 쉽다. 따라서 HIFU 치료 전 치료과정의 충분한 정보를 제공하여 불확실성을 감소시킬 수 있는 교육프로그램의 개발이 필요한데[7], 연구자가 개발한 본 동영상 교육프로그램이 HIFU 치료를 받는 자궁양성종양 환자들의 불확실성을 낮추는 데 유용하게 작용한 것으로 본다.

Jeon과 Park [18]은 본 연구의 대상자들과 치료방법은 달랐으나, 자궁절제술 환자를 대상으로 자궁절제술 전 동영상을 이용한 수술 전 교육이 자궁절제술 환자들의 불확실성을 낮추는 데 효과적이라고 보고하여 본 연구의 결과와 일치하였다. 척추 수술 환자와[19] 백내장 수술 환자를 대상으로 동영상 중심의 간호 교육을 실시한 연구에서 불확실성이 감소한 결과도 본 연구의 결과를 지지하였다[27]. 이러한 여러 연구들을 두고 볼 때, 연구 대상자의 치료법은 다르지만 동영상을 이용한 교육프로그램은 환자의 불확실성을 낮추는 데 효과적임을 알 수 있었다. 그러므로 연구자가 개발한 본 동영상 교육프로그램은 HIFU 치료를 받는 환자들의 불확실성을 낮추는 간호 중재로 유용하게 활용할 수 있을 것이다.

다음으로, 본 연구 대상자의 기분을 병원을 방문한 환자가 의사의 진료를 받기 위해 외래와 병실에서 기다리는 동안 가장 흔히 느끼는 환자 불안과 우울 기준[21]으로 조사한 결과, 본 연구의 동영상 교육프로그램은 HIFU 치료를 받는 자궁양성종양 환자의 기분을 개선하는 데 유용하였다. 이러한 결과는 수술 전 수술실 간호사의 동영상을 이용한 지지 간호 중재가 환자의 불안을 감소시킨 연구 결과와 일치하였다[14]. 또한 자궁절제술 전 동영상을 이용한 교육이 시간의 흐름에 따라 불안과 우울을 감소시킨 결과는 본 연구를 지지하였다[18].

환자들은 HIFU 치료 중 낯선 환경과 익숙하지 않은 치료과정으로 불안을 경험하며 이러한 불안을 완화시킬 수 없는 무기력함으로 우울을 경험한다[7]. 본 연구의 실험군은 구두 설명과 유인물을 이용한 주입과 암기 위주의 기존 교육과 달리, HIFU 치료 전 동영상 교육프로그램으로 교육을 받았다. 영상과 더불어 음향 효과를 통해 집중력을 높여 치료과정을 이해하고 치료 환경을 사전 체험하며, 정보를 습득하였다. 그러므로 HIFU 치료 중 유치도뇨관 유지 및 복부 압박, 고강도 초음파를 병변에 가할 때의 통증과 불편감 등이 HIFU 치료의 성공과 합병증 예방을 위해 반드시 필요한 중요 과정임을 충분히 숙지하여, 환자가 치료과정을 이해하고 수용하여 불안과 우울이 감소하였을 것으로 여겨진다.

한편, 본 연구 대상자의 동영상 교육프로그램은 HIFU 치료를 받는 자궁양성종양 환자의 자기 효능감을 향상시키는 데 효과적임을 알 수 있었다. 이러한 결과는 동영상을 이용한 어깨 운동 융합 프로그램에 참여한 어깨 수술 환자들의 자기 효능감이 높아진 결과와 유사하였다[15]. 어깨 수술과 HIFU 치료는 치료 방법과 부위는 다르나 치료 후 재활 치료와 HIFU 치료 후 자가간호를 잘 할 수 있다는 자신감은 치료의 효과를 높이는 유용한 전략이 될 수 있을 것이다. HIFU 치료 환자들은 자궁양성종양을 자신이 스스로 건강을 잘 돌보지 못하여 발생한 건강 문제로 인식하고 자책하였으나, 자신들의 건강 관리와 삶의 질에 영향을 미치는 결정 과정에 적극 참여하여야 함을 깨닫고, HIFU 치료를 받으며 그 방법과 과정에 관한 정보를 요구하고 건강 관리를 위한 강한 의지를 보였다[8]. 본 연구에 적용한 동영상 교육프로그램이 HIFU 치료방법, 준비과정 및 치료과정에 대한 정보 뿐만 아니라 HIFU 치료 후 나타날 수 있는 증상, 식이조절, 운동, 성생활 및 임신 가능 시기 등에 대한 정보를 제공하고, 동영상 시청 후 이해되지 않는 부분에 관하여 연구자가 질문을 받고 부연 설명을 하며 환자의 심리적 불안에 공감하면서, 환자 스스로 HIFU 치료 후 자가간호를 잘 수행할 수 있다는 자기 효능감을 증진시킨 것으로 여겨진다.

본 연구 대상자의 주 연령층은 50세 이하의 폐경기 이전 여성으로, 자궁절제술의 연구 대상자 평균 나이 49.3세[18]나 HIFU 치료 경험을 연구한 대상자의 32–48세[7]와 비슷한 결과를 보여, 자궁양성종양이 가임기와 중년 여성의 건강에 심각한 영향을 미치는 질환임을 알 수 있었다. 교육과 경제 수준은 중산층의 대졸 이상 고학력자가 선호하는 것으로 나타나 고졸의 비율이 가장 높은 자궁절제술보다 대졸 이상 고학력자의 비율이 높았다[18].

자궁양성종양 환자들에게 자궁절제 수술을 권유한 산부인과 의사들은 수술 방법을 설명했지만 HIFU 치료 방법을 설명한 사례는 매우 드물었다. 진단 후 산부인과 의사로부터 HIFU 치료법을 권유받지 못한 것은 HIFU 치료 전 환자들의 불확실성을 높이는 요인 중 하나가 될 수 있으므로 수술적 치료와 더불어 HIFU 치료에 대한 산부인과 전문의들의 자세한 설명과 정보 제공이 필요하다고 본다.

간호사는 환자의 보건교육자이고 옹호자이다[28]. 간호사에 의해 이루어지는, 이론과 실무에 기반하여 체계적으로 제공하는 간호 중재는 환자로 하여금 자신이 받는 치료법과 과정에 대하여 충분히 이해한 후 치료를 받게 하며, 치료과정에 적극적으로 참여하게 돕는다. 그러므로 의료기술의 발전과 더불어 간호 중재의 기법도 개발되어, 간호사의 교육을 받은 환자들이 높아진 자기 효능감으로 자신의 치료에 적극 참여할 수 있도록 지지하는 노력이 필요하다고 여겨진다.

이로써 자궁양성종양 환자를 대상으로 HIFU 치료를 잘 받게 하기 위한 동영상 교육프로그램은 환자의 불확실성, 기분 및 자기 효능감 등의 개선에 효과적임을 알 수 있었다. 본 연구자가 개발한 동영상 교육프로그램은 실제 경험하게 될 환경을 보다 더 현실적이고 역동적으로 감지하게 할 뿐만 아니라 저학력의 환자도 쉽게 이해할 수 있어, 환자들의 건강 증진과 치유에 유용하게 기여하는 간호 중재 프로그램이라고 볼 수 있겠다.

그러므로 자궁양성종양 환자들에게 기존의 표준화되지 않은 구두 설명 대신 항목별로 체계적인 틀을 갖춘 구조화된 정보 제공 교육프로그램을 적용하여 효율적인 실무이론의 개발에 기여한 점을 본 연구의 의의로 볼 수 있다.

그러나 본 연구는 창원시 한 지역 종합병원에 온 환자를 대상으로 실시한 연구이므로 일반화하기는 어려움이 따르며, 본 연구에 사용된 HIFU 기종은 엎드린 자세로 HIFU 치료를 진행하는 기종으로 앙와위(supine position)로 진행하는 기종의 HIFU 치료 중재와는 차이가 있어 프로그램의 적용에 제한이 있다. 그리고 마취 없이 진행되는 HIFU 치료의 특성상 치료 중 동반되는 통증에 대한 중재 효과를 연구하지 못한 아쉬움이 있다. 그러므로 추후 연구를 수행할 경우 앙와위로 진행하는 HIFU 치료의 간호 중재 개발과 치료 중 동반되는 통증을 완화시켜 주는 중재 연구를 제언한다.

동영상 교육프로그램은 인터넷과 휴대폰의 보급이 활성화된 우리나라 환경에서 모바일 어플리케이션(mobile application) 또는 온라인 공유 플랫폼 등을 통해 쉽게 제공할 수 있으며, 교육 대상자가 스스로 반복 학습하면서 교육의 효과를 상승시킬 수 있다. 따라서 다양한 분야의 간호 중재 프로그램의 개발이 필요하며 이는 여성건강 간호학의 발전에 기여할 것이라고 본다.

## Figures and Tables

**Table 1.** High-intensity focused ultrasound video education program

Themes	Contents	Time (second)
Introduction	Guide to the HIFU video education program	15
Function of the uterus	Therapeutic characteristics of benign uterine tumors	95
	Effects of hysterectomy on women's health	
Anatomy and physiology of the uterus	Anatomy and physiology of the uterus and female genitalia	78
	Uterine functions in the human body	
HIFU treatment	Principles of HIFU treatment	110
	Indications for HIFU treatment	
	Safety of HIFU treatment	
HIFU pre-test	Checking laboratory blood tests	60
	Checking chest X-rays	
	Checking abdominal MRI (enhancement)	
Preparation pre-HIFU	Shaving	90
	Enema	
	Foley catheterization	
HIFU treatment process	Position	32
	Communication	
	Pain management	
Precautions post-HIFU treatment	Vaginal discharge	120
	Bowel discomfort	
	Shower, exercise, sex life	
	Sitz bath, abdominal massage	
	Diet control	
	Contraception period	
Total		600

HIFU: high-intensity focused ultrasound; MRI: magnetic resonance imaging.

**Table 2.** Homogeneity test of general characteristics and disease-related characteristics between the two groups (N=54)

Variable	Categories	n (%)			χ^2^or t	*p*
		Total (n=54)	Experimental (n=27)	Control (n=27)		
Age (year)	≤39	12 (22.2)	9 (33.3)	3 (11.1)	4.06	.131
	40–49	34 (63.0)	14 (51.9)	20 (74.1)		
	50–­59	8 (14.8)	4 (14.8)	4 (14.8)		
Marital status	Married	43 (79.6)	19 (70.4)	24 (88.9)	2.85	.175
	Unmarried	11 (20.4)	8 (29.6)	3 (11.1)		
Educational level	≥University	37 (68.5)	21 (77.8)	16 (59.3)	2.15	.241
	≤High school	17 (31.5)	6 (22.2)	11 (40.7)		
Occupation	Yes	15 (27.8)	5 (18.5)	10 (37.0)	2.31	.224
	No	39 (72.2)	22 (81.5)	17 (63.0)		
Economic status	High	3 (5.6)	3 (11.1)	0 (0.0)	3.42	.181
	Middle	48 (88.9)	23 (85.2)	25 (92.6)		
	Low	3 (5.6)	1 (3.7)	2 (7.4)		
Diagnosis	Uterine myoma	38 (70.3)	19 (70.4)	19 (70.4)	0.26	.881
	Adenomyosis	9 (16.7)	5 (18.5)	4 (14.8)		
	Both	7 (13.0)	3 (11.1)	4 (14.8)		
Source for seeking disease-related information	Internet	38 (70.3)	16 (59.2)	22 (81.4)	3.20	.202
	Patients & other	13 (24.1)	9 (33.4)	4 (14.8)		
	Gynecologists	3 (5.6)	2 (7.4)	1 (3.8)		

**Table 3.** Comparison of uncertainty, anxiety, depression, and self-efficacy between the two groups (N=54)

Variable	Categories	Mean±SD		t	*p*
		Experimental (n=27)	Control (n=27)		
Uncertainty	Pre	88.03±14.65	89.37±8.14	0.41	.682
	Post	73.18±10.96	86.18±11.10	4.37	<.001
	Post-pre	14.85±17.47	3.19±9.66	4.33	<.001
Anxiety	Pre	15.80±4.03	13.8±3.47	–2.05	.044
	Post	11.82±4.40	13.46±2.86	3.93	<.001
	Post-pre	–4.44±4.33	0.11±1.21	–4.07	<.001
Depression	Pre	13.76±3.80	13.63±2.67	–.15	.876
	Post	11.58±3.30	13.16±3.05	3.00	<.004
	Post-pre	–2.67±3.55	–0.33±2.05	–3.55	<.001
Self-efficacy	Pre	41.85±4.24	40.44±5.10	–1.10	.276
	Post	46.40±4.69	40.92±4.48	–4.25	<.001
	Post-pre	–4.18±4.72	–0.51±2.95	–4.39	<.001
